# Both Dietary Supplementation with Monosodium L-Glutamate and Fat Modify Circulating and Tissue Amino Acid Pools in Growing Pigs, but with Little Interactive Effect

**DOI:** 10.1371/journal.pone.0084533

**Published:** 2014-01-21

**Authors:** Zemeng Feng, Xiaoli Zhou, Fei Wu, Kang Yao, Xiangfeng Kong, Tiejun Li, Francois Blachier, Yulong Yin

**Affiliations:** 1 Institute of Subtropical Agriculture, Chinese Academy of Sciences, Research Center of Healthy Breeding Livestock & Poultry, Hunan Engineering & Research Center of Animal & Poultry Science, Key Lab Agro-ecology Processing Subtropical Region, Scientific observational and experimental Station of Animal Nutrition and Feed Science in South-Central, Ministry of Agriculture, Changsha, Hunan, Peoples Republic of China; 2 College of Food and Pharmacy Engineering, Guiyang University, Guiyang, Guizhou, Peoples Republic of China; 3 Nutrition Physiology and Ingestive Behavior, Paris Institute of Technology for Life Food and Environmental Sciences, Paris, France; Rosalind Franklin University, United States of America

## Abstract

**Background:**

The Chinese population has undergone rapid transition to a high-fat diet. Furthermore, monosodium L-glutamate (MSG) is widely used as a daily food additive in China. Little information is available on the effects of oral MSG and dietary fat supplementation on the amino acid balance in tissues. The present study aimed to determine the effects of both dietary fat and MSG on amino acid metabolism in growing pigs, and to assess any possible interactions between these two nutrients.

**Methods and Results:**

Four iso-nitrogenous and iso-caloric diets (basal diet, high fat diet, basal diet with 3% MSG and high fat diet with 3% MSG) were provided to growing pigs. The dietary supplementation with fat and MSG used alone and in combination were found to modify circulating and tissue amino acid pools in growing pigs. Both dietary fat and MSG modified the expression of gene related to amino acid transport in jejunum.

**Conclusions:**

Both dietary fat and MSG clearly influenced amino acid content in tissues but in different ways. Both dietary fat and MSG enhance the absorption of amino acids in jejunum. However, there was little interaction between the effects of dietary fat and MSG.

## Introduction

As an umami flavor, monosodium L-glutamate (MSG) has been widely used as a food additive. The worldwide production and consumption of MSG has recently increased, and it is even expected to further increase in the next years (http://www.ihs.com/products/chemical/planning/ceh/monosodium-glutamate.aspx). MSG is considered as safe among food additives by the Joint Expert Committee on Food Additives of the United Nations Food and Agriculture Organization and World Health Organization [1]. When MSG is consumed, L-glutamate (Glu) is released in the small intestine lumen and metabolized notably by the enterocytes [2]. The regular daily consumption of MSG presumably modify the metabolism of nutrients, notably the metabolism of AAs in the body [3], and protein synthesis regulation and proteolysis [4], [5]. Only few studies have reported the effects of daily oral MSG supplementation on AA metabolism.

The Chinese population, including children and adolescents, has been undergoing a rapid transition to a high-fat diet. This change has occurred faster than that in western children over the last century [6], [7]. MSG is also currently widely added to food on a daily basis in China. Thus, it would be interesting to evaluate the metabolic effects of the combination of dietary fat and MSG. Pigs are considered to be a suitable animal model for studying human nutrition due to their apparent similarities with humans; i.e., both species are omnivorous, and they share nutritional and digestive characteristics [8]. In the present study, MSG was provided to growing pigs at a dose that was only slightly higher than that found in human food, without or with a plausible amount of dietary fat content. The aim of the present study was to determine the effect of dietary supplementation with MSG on the AA balance in tissues and to clarify whether the interaction between fat and MSG influence AA metabolism.

## Methods and Materials

### Experimental design, animals, and diets

Since rodents may be more sensitive to MSG than humans [9], the pig model was used in the present study. A total of 32 growing pigs (crossbred population composed of York, maternal Landrace, paternal Landrace, and Duroc breeds; average body weight 25±1.3 kg) from 4 litters in a pig farm located in Changsha were used. The pigs were randomly divided into four groups (8 animals per group with half males and half females). Pigs were raised in individual cages and provided with water and four different diets containing different amounts of fat and MSG (basal diet, high fat diet, basal diet with 3% MSG and high fat diet with 3% MSG). The basal diet group was used as control. According to previous experiments [10], the four diets were prepared to be iso-caloric and alanine was used to allow iso-nitrogenous content of all diets [11], [12]. The dose of MSG and dietary fat contents were designed according to the NRC 1998 and the consumption in human food. The detailed composition of the four diets are shown in **Table 1**. The total AAs in each diet were determined and the results are shown in **Table 2**. The four diets were provided to the pigs *ad libitum*. In the day time, natural light was provided, and in the dark period, fluorescent lamps were used and turned down at 8:00 pm. Water was provided freely during the entire experiment. Blood samples were firstly collected into 10-mL heparin-coated tubes, and then centrifuged (3,000×g for 10 min at 4°C). The supernatants (plasma) were immediately separated and stored at −70°C until analysis. The sampled pigs were sacrificed by jugular puncture under general anaesthesia via the intravenous injection of 4% sodium pentobarbital solution (40 mg/kg BW) and immediately eviscerated. Samples of the *longissimus dorsi*, liver, kidney and segments of the intestine including the duodenum, jejunum, ileum and colon (cleaned by ice-cold normal saline) were collected, immediately frozen in liquid nitrogen and stored at −70°C until analysis. All experimental procedures used in the present study were approved by the Animal Care and Use Committee of the Chinese Academy of Sciences [13].

**Table 1:** Composition of experimental diets.

Item	Basal diet (BD)	High fat diet (HF)	Basal diet + 3% MSG (BDM)	High fat diet + 3% MSG (HFM)
**Ingredient composition (%)**				
Corn	71.37	59.80	70.30	59.58
Soybean meal	19.20	21.27	16.80	21.50
Corn starch	0.00	7.00	0.00	5.00
Corn Gluten Meal	5.00	2.50	7.00	3.10
MSG	0.00	0.00	3.00	3.00
Alanine	1.58	1.58	0.00	0.00
L-Lysine monohydrochloride	0.15	0.15	0.20	0.12
Soybean oil	0.00	5.00	0.00	5.00
Premix^a^	2.70	2.70	2.70	2.70
**Calculated analysis**				
DE, Mj/kg	13.98	13.92	13.87	13.98
CP, %	17.93	17.88	17.95	17. 91
Fat, %	4.35	9.39	4.51	9.45
Ca, %	0.60	0.59	0.58	0.59
P, %	0.45	0.48	0.44	0.46

a Composition (%): CaHPO_4_, 27.78; Mountain flour, 24.07; NaCl, 11.11; Medical stone, 12.33; Powdered rice hulls, 18.81; FeSO_4_, 0.74; ZnSO_4_, 0.74; Selenium powder (1%), 0.15; Iodine powder (1%), 0.15; CuSO_4_, 0.37; MnSO_4_, 0.30; Choline chloride, 2.22; Growth pig multidimensional, 1.11; Antioxidants (Ethoxyquin 66%), 0.11.

**Table 2:** Analyzed AA composition of the experimental diets (%, as-fed basis).

Item	Treatment			
	BD*	HF*	BDM*	HFM*
**Indispensable AAs**				
Arginine (Arg)	1.25	1.00	1.01	1.01
Histidine (His)	1.28	1.32	1.06	1.04
Isoleucine (Ile)	0.80	0.63	0.61	0.65
Leucine (Leu)	1.82	1.70	1.49	1.56
Lysine (Lys)	0.73	0.60	0.56	0.55
Phenylalanine (Phe)	0.83	0.88	0.77	0.84
Threonine (Thr)	1.17	1.01	0.78	0.82
Tyrosine (Tyr)	0.49	0.54	0.52	0.54
Valine (Val)	0.96	0.84	0.76	0.75
**Dispensable AAs**				
Alanine (Ala)	2.26	1.90	0.82	0.91
Aspartate (Asp)	1.24	1.31	1.03	1.17
Glutamate(Glu)	2.53	2.55	3.61	4.09
Glycine (Gly)	0.77	0.88	0.73	0.83
Proline (Pro)	3.63	3.69	3.30	3.81
Serine (Ser)	1.16	1.27	0.88	0.98

* Abbreviations: BD, Basal diet; HF, High fat diet; BDM, Basal diet +3% monosodium L-glutamate; HFM, High fat diet +3% monosodium L-glutamate.

### Serum analyses

Plasma total concentrations of albumin, alanine transaminase, aspartate aminotransferase, γ-glutamyl transpeptidase, creatine kinase, total protein and serum urea were determined with an automatic analyzer (Beckman Instruments, Inc., Fullerton, CA) using reagents purchased from Beijing Leadman Biochemistry Co., LTD.

### Determining of AA concentrations using the stable isotope dilution HPLC–electrospray ionization (ESI)–MS/MS method

To determine contents of AAs in experimental diet, 5 g samples of diets were dissolved in hydrochloric acid solution (6 mol/L) at a concentration equal to 20 mg/ml, and hydrolized for 24 h, before centrifugation at 13200 rpm for 5 min. The supernatants were filtered with hydrophilic membrane, diluted 40 folds with methanol, and then were dried under nitrogen at 50°C. The samples were finally dissolved in 100 µl methanol solution (v/v = 1∶1) for the derivatization procedure. Ten µl of the extracted solution were transferred to a new centrifuge tube, and mixed with 90 µl of the stable isotope solution (the stable isotope being dissolved with methanol), vortexed and then centrifuged at 13,200 rpm for 5 min. The supernatants were transferred into a new centrifuge tube, dried under nitrogen at 50°C, mixed with 60 µl Hydrochloric acid/n-butyl alcohol derivative liquid, vortexed and then centrifuged at 13,200 rpm for 5 min. The supernatants were transferred into a new centrifuge tube and kept at 65°C for 15 min, dried under nitrogen at 50°C, and dissolved in 100 µl acetonitrile solution (v/v = 4∶1), vortexed and then centrifuged at 13,200 rpm for 5 min. The supernatants were used for the analysis of AAs by LC/mass spectrometer (HPLC Ultimate 3000, Dionex; 3200 Q TRAP LC-MS/MS, AB) with gas chromatography using a capillary column (AAA C18 5 µM 150×4.6 mm). The column temperature was 50°C. The sample size was 10 µl. The other parameters were as follows: moving phase (A: 1% formic acid solution, B: 1% formic acid dissolved with acetonitrile); CUR, 20.00; CAD, Medium; CXP, 5.00; IS, 5500.00; DP, 35.00; GS1, 55.00; GS2, 60.00; TEM, 580.00; EP, 10.00. The mass spectrometry detection gradient solutions were as follow: Step 0: time, 0 min; Flow rate, 800; A (%), 98; B (%), 2. Step 1: time, 10 min; Flow rate, 800; A (%), 72; B (%), 28. Step 2: time, 10.1 min; Flow rate, 800; A (%), 0; B (%), 100. Step 3: time, 16 min; Flow rate, 800; A (%), 0; B (%), 100. Step 4: time, 16.1 min; Flow rate, 800; A (%), 98; B (%), 2. Step 5: time, 25 min; Flow rate, 800; A (%), 98; B (%), 2. The determination of free AA concentrations in tissues was done using the method described above but without the steps of acidolysis. The whole processing was done in Beijing Amino Medical Research CO., LTD.

### Quantitative real-time reverse transcription-polymerase chain reaction

Total RNA was extracted from samples using TRIzol® Reagent (Invitrogen-Life Technologies, CA, USA) following the manufacturer's suggested protocol and finally dissolved in DEPC-treated water. Quality and concentration of the extracted RNA were checked by spectrophotometry using NanoDrop® ND2000 (NanoDrop Technologies Inc., DE, USA), respectively. Then, 1.0 µg of total sample RNA was incubated with DNase I (Fermentas), and reverse-transcribed with oligo dT and random primers using reverse transcription using First-Strand cDNA Synthesis Kit according to the manufacturer's instructions (TAKARA, Dalian, China). Finally, the cDNAs were stored at −70°C before further processing. qPCR was performed with an ABI PRISM 7900 HT (Applied Biosystems, USA) using 384-well plates. Duplicate sample analysis was routinely performed in a total volume of 10 µl using SYBR®Premix Ex TaqTM II (TAKARA, Dalian, China). The primers were designed using Primer 5.0 software and be listed in Table 3. Data were analyzed using the ABI 7900 SDS software (version 2.3, Applied Biosystems).

**Table 3:** Primers used in this study.

Gene	Gene Bank NO.	Sequence	Product length	Reference
β-ACTIN	XM_003357928.1	Sense: 5′ GGACTTCGAGCAGGAGATGG 3′Anti-sense: 5′ GCACCGTGTTGGCGTAGAGG 3′	233 bp	In this study
CaR	XM_003132642.1	Sense: 5′ GGGACCAGGAAAGGAATCAT 3′Anti-sense: 5′ CACGGCAAAGAGGGTGAGT 3′	219 bp	In this study
GPRC6A	XM_003480266.1	Sense: 5′ CTTGAGAAAATCATAGCAGAAGCC 3′Anti-sense: 5′ GGAATGGTAGTTATCTTGGTGGC 3′	161 bp	In this study
T1R1	XM_003356140.1	Sense: 5′ TCCCTGGGCTTCATACTGG 3′Anti-sense: 5′ TTCTCTGGCAAGTCCTTACCC 3′	92 bp	In this study
EAAC1	NM_001164649.1	Sense: 5′ GGCACCGCACTCTACGAAGCA 3′Anti-sense: 5′ GCCCACGGCACTTAGCACGA 3′	177 bp	In this study
LAT1	NM_001110421.1	Sense: 5′ TTTGTTATGCGGAACTGG 3′Anti-sense: 5′ AAAGGTGATGGCAATGAC 3′	155 bp	In this study
B^0,+^	NM_001110171.1	Sense: 5′ GAACCCAAGACCACAAATC 3′Anti-sense: 5′ ACCCAGTGTCGCAAGAAT 3′	180 bp	In this study
ASCT2	XM_003127238.1	Sense: 5′ GATTGTGGAGATGGAGGATGTGG 3′Anti-sense: 5′ TGCGAGTGAAGAGGAAGTAGATGA 3′	128 bp	In this study
PEPT1	NM_214347.1	Sense: 5′ CATCGCCATACCCTTCTG 3′Anti-sense: 5′ TTCCCATCCATCGTGACATT 3′	143 bp	In this study

### Statistical analyses

The data regarding gene expression were expressed as means ± SEM, and those regarding AA concentrations were expressed as means ± SD. Differences between groups were assessed by ANOVA. Statistical analyses were performed with SAS 9.2 (SAS Institute Inc., North Carolina, USA). The difference were considered as statistically significant at P<0.05.

## Results

### MSG and fat modify serum biochemical parameters

**Figure 1:**
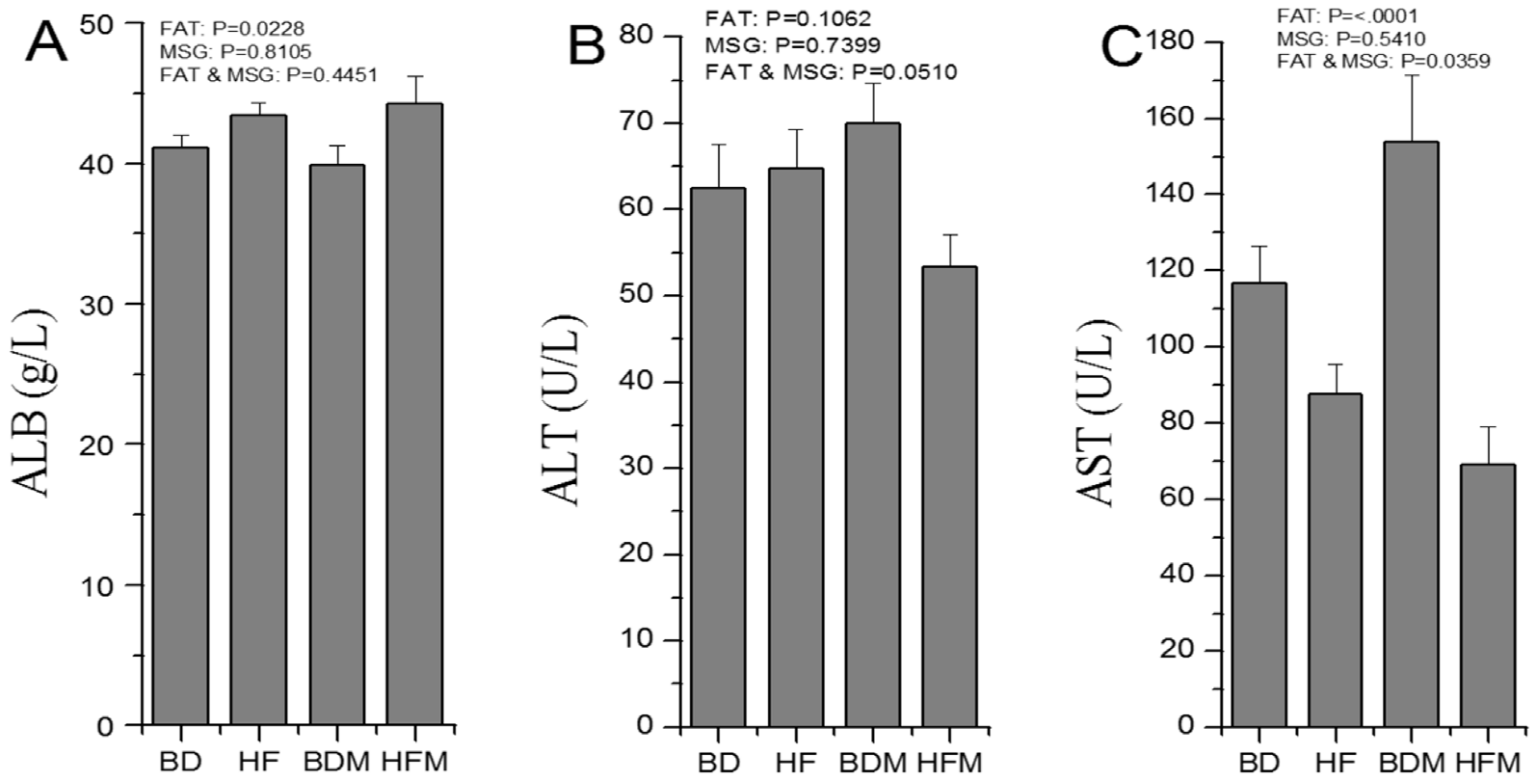
Dietary fat and MSG supplementation for one month effect the concentrations of serous biochemical parameters in growing pigs (n = 8). Abbreviation, ALB: Albumin, ALT: Alanine transaminase, AST: Aspartate aminotransferase.

Serum albumin was firstly tested, and the results are shown in **Fig. 1A**. Dietary fat clearly increased the serum albumin concentration (p = 0.0228), while MSG had no effect, and there was no interaction between the effect of dietary fat and MSG on serum albumin. To clarify the changes in AA metabolism, the activities of serum alanine transaminase and aspartate aminotransferase were measured, and the results are shown in **Fig. 1B** and **Fig. 1C**. Dietary fat significantly reduced the activity of aspartate aminotransferase (p<0.0001). Dietary fat and MSG synergistically reduced the activities of serum alanine transaminase (p = 0.0510) and aspartate aminotransferase (p = 0.0359). There were no significant differences between treatments for the other serum biochemical parameters, including creatine kinase, total protein and serum urea.

### MSG and fat modify serum AA profiles

Serum is the main circulating AA pool and the serum AA profile is often used as an index for the diagnosis of several diseases [14]. To determine the effects of fat and MSG on serum AAs, the AA profile was measured and the results are shown in **Table 4**. Dietary fat clearly reduced the concentrations of Thr (p = 0.0212), Val (p = 0.0246), Tyr (p = 0.0022), Cit (p = 0.0378), Gln (p = 0.0224), Orn (p = 0.0013) and Ala (p = 0.0100), while elevated the concentration of Gly (p = 0.0393). MSG obviously lessen the concentrations of 3-methyl-histidine (3MHis) (p = 0.0242), while increasing the concentrations of Hyp (p = 0.0322). Dietary fat and MSG had opposite effects on Cit (p<0.0001), Gln (p = 0.0004) and Pro (p = 0.0321). There were no changes in other AA concentrations, including Glu.

**Table 4:** Effect of dietary fat and MSG and their interaction on serous AA concentrations in growing pigs (n = 8).

AA	Measurements (Mean±SD) µmol/L				P values		
	BD*	HF*	BDM*	HFM*	Fat Effect	GSM Effect	Interaction
Arg	368.30±33.00	361.47±54.12	343.07±52.55	334.55±81.14	0.7482	0.2823	0.9718
His	226.3±17.30	210.00±28.99	225.93±31.45	231.80±34.08	0.6351	0.3746	0.3238
Ile	184.44±48.39	177.91±21.61	176.88±16.10	166.29±30.45	0.5148	0.4659	0.8766
Leu	361.26±82.58	339.21±31.51	368.51±51.99	314.35±53.01	0.1215	0.7125	0.5034
Lys	251.77±29.66	209.05±32.41	226.94±29.21	222.34±44.08	0.0810	0.7419	0.1725
Phe	108.70±10.63	105.89±5.34	115.07±17.24	111.77±14.81	0.6064	0.2308	0.9610
Thr	115.74±32.51	85.94±20.59	107.07±18.03	85.81±23.18	**0.0212**	0.6275	0.6916
Trp	34.96±3.80	37.34±3.80	41.28±6.25	36.15±6.65	0.3976	0.1982	0.0919
Val	362.01±86.70	294.35±32.44	409.11±58.13	317.01±55.94	**0.0046**	0.1793	0.6311
Gly	908.97±98.31	1007.76±146.57	924.94±145.06	1080.74±195.79	**0.0393**	0.4701	0.6169
Ser	168.48±19.04	166.87±18.36	166.73±23.31	151.33±33.34	0.3672	0.3932	0.4718
Tyr	201.09±26.10	167.63±24.32	199.69±39.08	147.41±32.36	**0.0022**	0.3516	0.4429
Asn	108.42±11.86	110.92±22.43	113.86±14.29	115.57±26.15	0.5296	0.7860	0.9597
Asp	26.33±3.69	25.46±3.49	31.13±8.26	23.88±2.81	0.0625	0.4439	0.1369
Cit	116.59±10.90	76.77±11.35	86.18±4.11	111.73±9.25	**0.0378**	0.8562	**<0.0001**
Glu	420.64±45.12	413.02±19.81	453.14±45.68	417.33±77.27	0.3112	0.3890	0.5078
Gln	79.15±7.49	57.04±5.88	68.02±10.06	74.34±8.12	**0.0224**	0.5778	**0.0004**
Orn	173.79±20.70	148.24±9.78	206.57±39.09	157.14±19.39	**0.0013**	0.0515	0.0515
Cys	1.65±0.50	1.59±0.85	1.17±0.69	1.40±0.77	0.7198	0.2690	0.6172
Ala	810.03±106.61	625.86±161.16	741.42±88.53	662.43±86.93	**0.0100**	0.6972	0.2616
Hyp	433.80±126.73	309.90±61.85	461.44±74.49	444.31±65.65	0.0589	**0.0322**	0.1451
1MHis	4.87±0.90	4.59±0.77	4.62±0.58	4.99±0.56	0.9074	0.8509	0.2741
3MHis	7.93±0.68	9.02±1.72	7.23±1.63	6.96±1.52	0.5196	**0.0242**	0.2291
Pro	508.47±75.95	539.44±110.13	584.58±67.54	479.85±55.18	0.2837	0.6557	**0.0321**

* Abbreviations: BD, Basal diet; HF, High fat diet; BDM, Basal diet +3% monosodium L-glutamate; HFM, High fat diet +3% monosodium L-glutamate.

### Dietary supplementation with both fat and MSG can influence the expression of AA-sensing genes

**Figure 2:**
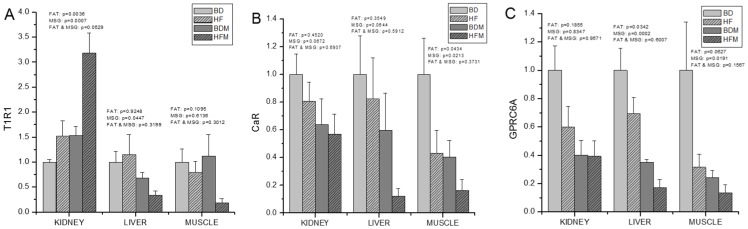
Dietary fat and MSG supplementation changed the express profiles of free AA-sensing genes, T1R1 (A), CaR (B) and GPRC6A (C) (n = 8). The results were normalized by the expression of β-actin. Abbreviations, T1R1: Taste receptor type 1 member 1, CaR: Ca^2+^-sensing receptor and GPRC6A: G-protein-coupled receptor family C member 6A.

To document the effects of dietary fat and MSG on AA-related gene expression, the expression of a broad spectrum of AA-sensing genes in the kidney, liver and muscle were determined. The results are shown in **Fig. 2**. Dietary fat can significantly up-regulated the expression of taste receptor type 1 member 1 (T1R1) in the kidney (p = 0.0036), and down-regulated its expression in muscle (p = 0.0434), and G-protein-coupled receptor family C member 6A (GPRC6A) in the liver (p = 0.0342). MSG significantly up-regulated the expression of T1R1 in the kidney (p = 0.0007), and down-regulated the expression of T1R1 in the liver (p = 0.0447). MSG also down-regulated the expression of extracellular Ca^2+^-sensing receptor (CaR) in the muscle (p = 0.0213), and significantly down-regulated the expression of GPRC6A in the liver (p = 0.002) and muscle (p = 0.0191).

### Dietary supplementation with fat and MSG modify AA pools in liver and muscle

The free AA profiles in the liver and muscle were determined, and the results are shown in **Table 5** and **Table 6**. Dietary fat increased the concentrations of Ser (p = 0.0078), Tyr (p = 0.0046), Asn (p = 0.0347), and Cys (p = 0.0493) in the liver, and also increased the concentrations of Gly (p = 0.0155) and Ala (p = 0.0441) in the muscle. MSG had no effect on AA pool in the liver and muscle. Dietary fat and MSG had opposite effects on the concentrations of Cit (p = 0.0004) in the liver and Hyp (p = 0.0092) in the muscle. Interestingly, while dietary fat and MSG each tended to reduce homocysteine (Hcy) in muscle, their combination increased the concentration of Hcy in muscle (p = 0.0258).

**Table 5:** Effect of dietary fat and MSG and their interaction on free AA concentrations in liver of growing pigs (n = 8).

AA	Measurements (Mean±SD) µmol/g				P values		
	BD*	HF*	BDM*	HFM*	Fat Effect	GSM Effect	Interaction
Arg	0.1327±0.0625	0.1207±0.0163	0.1282±0.0543	0.1799±0.0569	0.4508	0.3029	0.2347
His	1.0052±0.1216	1.1551±0.1327	0.9871±0.1716	1.0373±0.1093	0.1661	0.3367	0.4769
Ile	0.3963±0.0789	0.4216±0.0714	0.4013±0.0890	0.4540±0.0610	0.3237	0.6316	0.7240
Leu	1.1294±0.2158	1.2604±0.2027	1.2220±0.3047	1.2276±0.1119	0.5456	0.7903	0.5787
Lys	0.6742±0.1122	0.6991±0.0956	0.7286±0.2360	0.7209±0.0581	0.9057	0.6014	0.8221
Met	0.4915±0.0869	0.6358±0.1377	0.6213±0.1634	0.6552±0.0423	0.1546	0.2275	0.3652
Phe	0.6607±0.0572	0.7973±0.1195	0.7074±0.1360	0.7396±0.0616	0.1168	0.9139	0.3163
Thr	0.7792±0.1203	0.7580±0.1297	0.7707±0.1005	0.7800±0.0310	0.8349	0.9740	0.8457
Trp	0.7012±0.0579	0.6848±0.0579	0.6366±0.0689	0.6981±0.0961	0.5427	0.4896	0.3002
Val	0.7513±0.1277	0.7403±0.1243	0.8316±0.2750	0.7751±0.0637	0.6931	0.5037	0.7899
Gly	8.1919±1.0939	8.2064±0.7118	7.5550±0.8290	8.1158±0.7070	0.5113	0.4088	0.5325
Ser	1.3655±0.0725	1.6652±0.2807	1.2590±0.2177	1.5622±0.1080	**0.0078**	0.2897	0.9856
Tau	0.0064±0.0015	0.0053±0.0014	0.0083±0.0030	0.0063±0.0014	0.1423	0.1647	0.7110
Tyr	0.7390±0.0815	0.8699±0.1010	0.7633±0.0537	0.8872±0.0428	**0.0046**	0.5813	0.9249
Asn	1.1393±0.2034	1.3602±0.1799	1.2127±0.1462	1.3881±0.1252	**0.0347**	0.5543	0.7889
Asp	0.3810±0.0106	0.3913±0.0182	0.3407±0.0459	0.4145±0.0669	0.0678	0.6910	0.1561
Cit	0.0472±0.0073	0.0338±0.0036	0.0276±0.0067	0.0468±0.0085	0.4078	0.3386	**0.0004**
Glu	0.8486±0.0750	0.8031±0.0639	0.7822±0.0739	0.8632±0.1003	0.6634	0.9374	0.1373
Gln	4.0030±0.5849	3.8702±0.6666	4.2568±1.5683	4.2900±0.3983	0.9158	0.4793	0.8602
Orn	1.3427±0.2769	1.3010±0.0676	1.2007±0.1506	1.2474±0.1450	0.9783	0.2899	0.6257
Cys	0.6053±0.2061	1.1140±0.5901	0.4992±0.3967	0.9324±0.4406	**0.0493**	0.5168	0.8638
Hcy	0.0208±0.0022	0.0208±0.0023	0.0244±0.0071	0.0230±0.0030	0.7421	0.1940	0.7492
Abu	0.0821±0.0352	0.0693±0.0130	0.0773±0.0274	0.0866±0.0473	0.9192	0.7125	0.5175
Ala	2.8986±0.4294	2.8738±0.5048	2.3693±0.2375	2.7685±0.7133	0.4694	0.2294	0.4140
Car	0.0075±0.0051	0.0096±0.0032	0.0128±0.0037	0.0094±0.0013	0.7192	0.1809	0.1488
Hyp	0.4738±0.0911	0.4424±0.0834	0.4089±0.1271	0.3948±0.1804	0.7256	0.3912	0.8938
1MHis	0.0555±0.0058	0.0543±0.0023	0.0701±0.0238	0.0640±0.0129	0.6078	0.1056	0.7258
3MHis	0.0028±0.0009	0.0028±0.0006	0.0028±0.0011	0.0028±0.0005	0.9767	0.9979	0.9557
Pro	1.9254±0.4203	1.8970±0.3312	1.9873±0.2364	1.9755±0.2394	0.9009	0.6646	0.9588

* Abbreviations: BD, Basal diet; HF, High fat diet; BDM, Basal diet +3% monosodium L-glutamate; HFM, High fat diet +3% monosodium L-glutamate.

**Table 6:** Effect of dietary fat and MSG and their interaction on free AA concentrations in muscle of growing pigs (n = 8).

AA	Measurements (Mean±SD) µmol/g				P values		
	BD*	HF*	BDM*	HFM*	Fat Effect	GSM Effect	Interaction
Arg	0.1625±0.0422	0.2648±0.0861	0.1638±0.0378	0.2579±0.1319	0.0509	0.9463	0.9295
His	5.6863±0.4497	5.1265±1.4013	4.5657±0.7933	5.1890±0.6269	0.8192	0.3341	0.2436
Ile	0.0493±0.0429	0.0962±0.0306	0.0692±0.0032	0.0816±0.0257	0.1586	0.3939	0.6978
Leu	0.1501±0.0451	0.2082±0.0578	0.1749±0.0143	0.2132±0.0473	0.0668	0.5482	0.6750
Lys	0.1315±0.0688	0.1943±0.1379	0.1092±0.0341	0.1854±0.0738	0.1494	0.7484	0.8861
Met	0.0755±0.0193	0.0631±0.0174	0.0612±0.0247	0.0916±0.0406	0.4978	0.5555	0.1661
Phe	0.8512±0.2283	0.8399±0.1903	0.7747±0.1740	0.9901±0.2691	0.3603	0.6941	0.3384
Thr	0.1890±0.0285	0.1796±0.0457	0.1835±0.0588	0.2010±0.0897	0.8915	0.7862	0.6858
Trp	0.0792±0.0180	0.2138±0.1812	0.1138±0.0606	0.1111±0.0357	0.2537	0.4723	0.2212
Val	0.1394±0.0214	0.1770±0.0158	0.1487±0.0203	0.1539±0.0338	0.1233	0.5256	0.2219
Gly	1.3442±0.3177	2.5352±0.6128	1.3873±0.4196	1.9597±0.8191	**0.0155**	0.3663	0.3378
Ser	0.0957±0.0254	0.1504±0.0804	0.1133±0.0384	0.1220±0.0423	0.2871	0.7981	0.4188
Tau	0.0092±0.0021	0.0096±0.0035	0.0085±0.0026	0.0102±0.0031	0.4764	0.9876	0.6876
Tyr	0.1120±0.0213	0.1435±0.0142	0.1300±0.0210	0.1446±0.0475	0.2665	0.7228	0.7587
Asn	0.0736±0.0127	0.1130±0.0773	0.0836±0.0198	0.0860±0.0270	0.3991	0.6750	0.4402
Asp	0.0211±0.0055	0.0336±0.0174	0.0309±0.0115	0.0299±0.0181	0.5162	0.7433	0.3947
Cit	0.0615±0.0250	0.1068±0.0628	0.0775±0.0170	0.0701±0.0263	0.3865	0.5441	0.2118
Glu	0.0969±0.0201	0.2595±0.2180	0.1383±0.0753	0.1295±0.0488	0.2714	0.4436	0.2089
Gln	0.7110±0.3139	0.7705±0.0907	0.6290±0.1048	1.3935±0.7380	0.0773	0.1937	0.1303
Orn	0.0803±0.0332	0.1423±0.0394	0.0885±0.0504	0.1324±0.0672	0.0705	0.9543	0.7373
Hcy	0.0243±0.0058	0.0208±0.0062	0.0154±0.0021	0.0261±0.0061	0.1493	0.6501	**0.0258**
Abu	0.0252±0.0071	0.0397±0.0169	0.0280±0.0054	0.0532±0.0372	0.1090	0.4616	0.6480
Ala	2.8870±0.6440	3.8679±0.5953	2.8727±0.5401	3.4975±0.8887	**0.0441**	0.5730	0.6265
Car	2.7300±0.1985	2.6436±0.8156	2.2378±0.4714	3.1036±0.6674	0.2046	0.9493	0.1611
Hyp	4.5922±0.3088	6.1164±0.7317	5.4785±0.7462	5.1129±0.2452	0.1113	0.6689	**0.0092**
1MHis	0.7288±0.1544	0.8554±0.3061	0.6892±0.1155	0.8374±0.2182	0.2351	0.8069	0.9252
3MHis	0.0538±0.0029	0.0537±0.0066	0.0498±0.0083	0.0658±0.0145	0.1235	0.3587	0.1302
Pro	0.1387±0.0541	0.2443±0.0602	0.1902±0.0572	0.1897±0.0503	0.1179	0.8482	0.0944

* Abbreviations: BD, Basal diet; HF, High fat diet; BDM, Basal diet +3% monosodium L-glutamate; HFM, High fat diet +3% monosodium L-glutamate.

### The free AA pool in the kidney is markedly altered by supplementation with MSG

The kidney plays a major role in the homeostasis of AA pools leading us to determine the free AA profile in this tissue. The results are shown in **Table 7**. Dietary fat had little effect on the free AA pool in the kidney, and significantly reduced only the Car concentration (p = 0.0257). MSG significantly increased the concentrations of His (p = 0.0086), Ile (p = 0.0080), Leu (p = 0.0040), Met (p = 0.0004), Thr (p = 0.0038), Val (p = 0.0007), Ser (p = 0.0213), Tyr (p = 0.0194) and Ala (p = 0.0093) in the kidney. Dietary fat enhances the effects of MSG on the renal concentrations of His (p = 0.0309) and Ser (p = 0.0353), and weakens the effects of MSG on the renal concentrations of Phe (p = 0.0191) and Glu (p = 0.0316).

**Table 7:** Effect of dietary fat and MSG and their interaction on free AA concentrations in the kidney of growing pigs (n = 8).

AA	Measurements (Mean±SD) µmol/g				P values		
	BD*	HF*	BDM*	HFM*	Fat Effect	GSM Effect	Interaction
Arg	0.9470±0.3111	0.7981±0.0820	0.9481±0.1123	1.0189±0.1422	0.6799	0.2525	0.2569
His	0.6691±0.1667	0.5335±0.0496	0.7075±0.1089	0.8434±0.0854	0.9979	**0.0086**	**0.0309**
Ile	0.5304±0.1158	0.4364±0.0598	0.6663±0.1484	0.6465±0.0918	0.3164	**0.0080**	0.5089
Leu	1.3014±0.2530	1.1243±0.1220	1.6947±0.4033	1.6257±0.1118	0.3481	**0.0040**	0.6756
Lys	0.8814±0.2154	0.5983±0.0896	0.8761±0.1650	0.9079±0.1702	0.1567	0.0922	0.0826
Met	0.8680±0.2067	0.6548±0.0743	1.1503±0.2656	1.2547±0.1046	0.5570	**0.0004**	0.1032
Phe	1.1766±0.1318	1.0300±0.1052	1.0678±0.0525	1.1884±0.0884	0.7975	0.6244	**0.0191**
Thr	0.8093±0.1059	0.7107±0.0375	1.0033±0.1970	0.9747±0.1181	0.3396	**0.0038**	0.5939
Trp	0.7526±0.1921	0.5886±0.0499	0.6731±0.0896	0.7382±0.0529	0.3952	0.5433	0.0634
Val	0.9515±0.1755	0.7763±0.0779	1.1244±0.1325	1.1519±0.0723	0.2499	**0.0007**	0.1228
Gly	9.4363±1.9458	10.5489±1.9435	10.5423±1.2747	11.7677±1.1523	0.1749	0.1772	0.9457
Ser	1.7530±0.5085	1.3867±0.1570	1.7950±0.2530	2.1508±0.1540	0.9730	**0.0213**	**0.0353**
Tau	0.0081±0.0023	0.0066±0.0024	0.0080±0.0015	0.0064±0.0004	0.1283	0.8243	0.9759
Tyr	0.8383±0.1475	0.6935±0.1395	0.9365±0.1931	1.0319±0.1617	0.7650	**0.0194**	0.1633
Asn	1.2405±0.3099	1.0318±0.2082	1.3488±0.2205	1.4050±0.1131	0.5091	0.0527	0.2601
Asp	0.4706±0.0688	0.4303±0.0607	0.6204±0.0933	0.7142±0.0957	0.5212	**0.0002**	0.1240
Cit	0.0628±0.0151	0.0554±0.0112	0.0793±0.0135	0.0733±0.0213	0.4092	**0.0488**	0.9351
Glu	0.9042±0.2090	0.7012±0.0522	0.8124±0.0796	0.9003±0.0667	0.3543	0.3869	**0.0316**
Gln	6.5259±1.7905	4.5891±0.7030	6.8564±1.9451	6.0344±1.1389	0.0873	0.2538	0.4663
Orn	0.8699±0.0395	0.7463±0.1696	1.0106±0.0727	0.9056±0.2414	0.1614	0.0739	0.9052
Cys	1.3090±0.3987	1.0198±0.2387	0.9840±0.1491	1.1123±0.3494	0.6016	0.4537	0.1895
Hcy	0.0309±0.0038	0.0253±0.0031	0.0316±0.0059	0.0303±0.0043	0.1411	0.2218	0.3508
Abu	0.3091±0.0263	0.2770±0.0675	0.3468±0.0587	0.3103±0.0808	0.2882	0.2723	0.9451
Ala	3.2054±0.3749	3.3173±0.3605	3.9954±0.5641	3.7758±0.2491	0.7937	**0.0093**	0.4272
Car	0.0162±0.0040	0.0104±0.0037	0.0206±0.0086	0.0123±0.0044	**0.0257**	0.2764	0.6604
Hyp	0.4988±0.1545	0.4851±0.1848	0.4316±0.1025	0.4100±0.1874	0.8300	0.3940	0.9620
1MHis	0.1959±0.1090	0.1877±0.0668	0.2080±0.0604	0.2183±0.0625	0.9786	0.5907	0.8146
3MHis	0.0057±0.0025	0.0053±0.0004	0.0052±0.0007	0.0049±0.0020	0.6686	0.5875	0.8731
Pro	2.8357±0.4855	2.5139±0.6752	3.3902±0.7130	3.2030±0.4328	0.4044	0.0564	0.8231

* Abbreviations: BD, Basal diet; HF, High fat diet; BDM, Basal diet +3% monosodium L-glutamate; HFM, High fat diet +3% monosodium L-glutamate.

### Both dietary fat and MSG can modify the expression of the AA transporters in the jejunum

**Figure 3:**
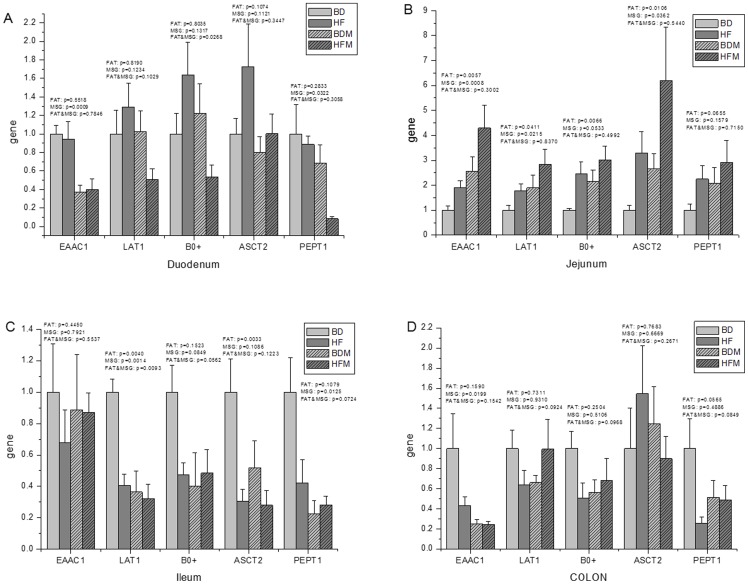
Dietary fat and MSG supplementation changed the express profiles of AA and peptide transporters in intestine: Duodenum (A), Jejunum (B), Ileum (C) and Colon (D) (n = 8). The results were normalized by β-actin. Abbreviation, EAAC1: Excitatory amino-acid carrier 1, LAT1: L-type AA transporter 1, B^0+^: B^0+^ AA transporter, ASCT2: ASC-like Na^+^-dependent neutral AA transporter 2 and PEPT1: Intestinal H^+^/peptide co-transporter.

To better understand AA absorption, the expressions of AA transporters in different intestinal segments was measured, and the results are shown in Fig. 3. These AA transporters include: Excitatory amino-acid carrier 1, EAAC1, which is the most abundant Glu and Asp transporter in the intestine; L-type AA transporter 1 (LAT1) and B^0+^ AA transporter (B^0+^), which can transport neutral and alkaline AAs; ASC-like Na^+^-dependent neutral AA transporter 2 (ASCT2), which can transport neutral AAs; and intestinal H^+^/peptide co-transporter (PEPT1), which mainly transports peptides in the intestine. While dietary fat had no effect on the expression of AA transporters, MSG significantly reduced the expression of EAAC1 (p = 0.0009) in the duodenum. The expression of B^0+^ in the duodenum was synergistically down-regulated by dietary fat and MSG (p = 0.0268). In the jejunum, dietary fat significantly increased the expression of EAAC1 (p = 0.0057), LAT1 (p = 0.0411), B^0+^ (p = 0.0066) and ASCT2 (p = 0.0106), while MSG enhanced the expression of EAAC1 (p = 0.0008), LAT1 (p = 0.0215), and ASCT2 (p = 0.0362). There was no interaction between the effects of dietary fat and MSG on the expression of AA transporters in the jejunum. In the ileum, dietary fat reduced the expression of LAT1 (p = 0.0040) and ASCT2 (p = 0.0033), while MSG reduced the expression of LAT1 (p = 0.0014) and PEPT1 (p = 0.0125). Dietary fat and MSG had synergistic effects on the expression of LAT1 (p = 0.0093). Although the colonic epithelial cells do not transport AAs, except in the neonatal period, amino acid transporter expression in the large intestine was also measured. Dietary fat had no effect on the expression of AA transporters in the colon, while MSG significantly reduced the expression of EAAC1 (p = 0.0199). To determine the consequence of the altered expression of AA transporters, free AA concentrations were measured. The disturbed AA balance in intestinal wall (**Tables 8** **–** **10**) suggests that dietary fat and MSG may modify the absorption and metabolism of AAs in the jejunum mucosa.

**Table 8:** Effect of dietary fat and MSG and their interaction on free AA concentrations in the duodenum of growing pigs (n = 8).

AA	Measurements (Mean±SD) µmol/g				P values		
	BD*	HF*	BDM*	HFM*	Fat Effect	GSM Effect	Interaction
Arg	0.8846±0.1367	0.7097±0.1640	0.7525±0.1574	0.6874±0.1975	0.1967	0.3538	0.5664
His	0.3046±0.0210	0.3024±0.0480	0.3227±0.0776	0.3052±0.0478	0.7519	0.6910	0.8025
Ile	0.2694±0.0270	0.3595±0.0748	0.2960±0.0694	0.2948±0.0823	0.3616	0.7875	0.2391
Leu	0.7975±0.0734	0.9642±0.1791	0.8155±0.1410	0.7860±0.2040	0.6471	0.4859	0.2766
Lys	0.7229±0.1480	0.5414±0.1231	0.6232±0.2073	0.4996±0.0768	0.0787	0.3762	0.7387
Met	0.4428±0.0547	0.4713±0.0753	0.4339±0.0870	0.3806±0.1118	0.5694	0.4003	0.4177
Phe	0.9920±0.0629	1.0577±0.0378	0.9670±0.0774	1.0587±0.1337	0.1441	0.7790	0.8061
Thr	0.5293±0.0597	0.5315±0.0901	0.5484±0.1140	0.4721±0.0749	0.3385	0.7997	0.4355
Trp	0.6741±0.0770	0.6070±0.0079	0.6276±0.1181	0.6653±0.0343	0.9283	0.8970	0.2787
Val	0.5238±0.0849	0.5087±0.0390	0.5781±0.1052	0.4881±0.1383	0.3538	0.6852	0.5441
Gly	5.1799±0.2854	4.2509±0.4071	4.8394±0.7070	5.0408±0.7832	0.4987	0.7460	0.1297
Ser	1.0356±0.0902	0.9338±0.2577	1.0818±0.3422	0.9192±0.2074	0.3216	0.8709	0.8248
Tau	0.0082±0.0020	0.0064±0.0004	0.0065±0.0018	0.0070±0.0014	0.5380	0.3977	0.2392
Tyr	0.4923±0.0570	0.5040±0.1507	0.4469±0.1118	0.3879±0.0981	0.4419	0.2060	0.5388
Asn	0.6531±0.0456	0.8194±0.2039	0.6251±0.1138	0.5776±0.1130	0.7580	0.0935	0.1179
Asp	0.2602±0.0417	0.2321±0.0099	0.2554±0.0637	0.27204±0.0112	0.9676	0.5835	0.3655
Cit	0.2171±0.0480	0.1505±0.0404	0.2059±0.0289	0.1743±0.0459	0.0724	0.9059	0.4690
Glu	0.8416±0.0889	0.7514±0.0126	0.7617±0.1445	0.8063±0.0321	0.7952	0.6276	0.2316
Gln	5.2678±1.1395	3.3681±0.5563	3.8318±2.0290	2.9714±0.7052	0.0742	0.1957	0.5047
Orn	0.2860±0.0282	0.2402±0.0548	0.2660±0.0614	0.2120±0.0365	0.0506	0.3726	0.8739
Cys	0.4577±0.2494	0.4586±0.0528	0.4052±0.2292	0.1950±0.1143	0.1916	0.2320	0.3592
Hcy	0.0324±0.0083	0.0275±0.0038	0.0296±0.0058	0.0229±0.0039	0.0718	0.3216	0.7919
Abu	0.4467±0.2345	0.4229±0.1655	0.2714±0.0624	0.2292±0.1940	0.5124	0.0905	0.9278
Ala	2.0632±1.3648	2.5732±0.7331	1.0084±0.2518	1.1637±0.2711	0.8274	**0.0222**	0.7030
Car	ND	ND	ND	0.0023±0.0046	-	-	-
Hyp	1.2359±0.1014	1.1934±0.2419	1.1894±0.1548	1.1761±0.1428	0.7100	0.6789	0.8645
1MHis	0.0608±0.0087	0.0493±0.0021	0.0636±0.0111	0.0611±0.0157	0.4389	0.3384	0.5014
3MHis	0.0019±0.0015	0.0020±0.0003	0.0023±0.0003	0.0033±0.0010	0.1869	0.2036	0.4324
Pro	1.1299±0.1135	1.2034±0.4353	1.1805±0.3533	0.8084±0.1438	0.1533	0.3872	0.1532

* Abbreviations: BD, Basal diet; HF, High fat diet; BDM, Basal diet +3% monosodium L-glutamate; HFM, High fat diet +3% monosodium L-glutamate.

**Table 9:** Effect of dietary fat and MSG and their interaction on free AA concentrations in the jejunum of growing pigs (n = 8).

AA	Measurements (Mean±SD) µmol/g				P values		
	BD*	HF*	BDM*	HFM*	Fat Effect	GSM Effect	Interaction
Arg	0.9768±0.1600	0.8492±0.1725	0.6560±0.1042	0.9141±0.5390	0.7151	0.5720	0.2928
His	0.6561±0.1010	0.5208±0.1103	0.3966±0.0644	0.6853±0.4396	0.5884	0.9017	0.1533
Ile	0.4385±0.1167	0.3659±0.1328	0.2563±0.0454	0.4349±0.2899	0.6043	0.7013	0.2336
Leu	1.1097±0.2204	1.0109±0.3390	0.7045±0.1153	1.0974±0.6867	0.5444	0.6040	0.3189
Lys	0.6884±0.2000	0.5124±0.1449	0.3926±0.1101	0.6086±0.3735	0.8814	0.5909	0.1647
Met	0.6478±0.0579	0.5336±0.1293	0.4855±0.1311	0.7460±0.4077	0.5892	0.6980	0.1837
Phe	1.5511±0.2391	1.3891±0.1835	1.0868±0.2235	1.3521±0.2060	0.6590	0.0788	0.0894
Thr	0.5060±0.1187	0.4752±0.1338	0.4465±0.0563	0.5832±0.2495	0.5679	0.6912	0.3717
Trp	0.9805±0.1634	0.8243±0.1009	0.7504±0.0078	1.0201±0.4854	0.7165	0.9313	0.1910
Val	0.6588±0.1331	0.6201±0.1207	0.4387±0.0720	0.7194±0.4921	0.4510	0.8104	0.3251
Gly	7.2757±1.4304	7.3174±0.6225	6.0244±0.1362	8.5587±3.8171	0.3066	0.8866	0.3217
Ser	1.2290±0.0487	1.0721±0.1713	0.8200±0.1462	1.3891±0.8955	0.4664	0.9830	0.2120
Tau	0.0093±0.0019	0.0083±0.0012	0.0085±0.0012	0.0084±0.0014	0.4450	0.6777	0.6002
Tyr	0.6192±0.0511	0.5275±0.1390	0.4557±0.1132	0.6867±0.4375	0.6272	0.8820	0.2730
Asn	1.3506±0.1797	1.1752±0.3555	0.7827±0.1188	1.4689±0.9654	0.4274	0.8098	0.1932
Asp	0.3615±0.0094	0.3158±0.0462	0.2774±0.0397	0.4028±0.2375	0.5931	0.8516	0.2637
Cit	0.2583±0.0226	0.2316±0.0743	0.1901±0.0559	0.2432±0.1289	0.7815	0.6332	0.4100
Glu	1.0359±0.1784	0.8849±0.0988	0.7696±0.0264	1.0561±0.4850	0.6670	0.9160	0.1826
Gln	5.5077±1.4716	4.2252±1.3554	3.3146±0.6840	5.0072±2.8550	0.8437	0.6337	0.1730
Orn	0.3249±0.0188	0.3521±0.0950	0.2210±0.0236	0.3429±0.2298	0.3368	0.5115	0.5363
Cys	0.6648±0.4137	1.0199±0.5695	0.6940±0.1640	0.7729±0.7343	0.4790	0.6692	0.6498
Hcy	0.0299±0.0040	0.0240±0.0028	0.0265±0.0037	0.0269±0.0027	0.1439	0.9327	0.1026
Abu	0.6243±0.1712	0.5643±0.1573	0.3208±0.0478	0.5413±0.3368	0.5119	0.2484	0.2621
Ala	2.8043±1.7161	2.1952±0.8293	1.1568±0.2406	2.1835±1.2608	0.7400	0.2665	0.2110
Car	0.0017±0.0030	0.0019±0.0038	0.0014±0.0024	0.0022±0.0043	-	-	-
Hyp	0.9298±0.1734	1.1109±0.1365	1.1292±0.1448	1.1767±0.1812	0.2155	0.1811	0.4578
1MHis	0.0455±0.0175	0.0448±0.0048	0.0578±0.0138	0.0720±0.0419	0.6317	0.1528	0.5947
3MHis	0.0023±0.0003	0.0019±0.0007	0.0021±0.0001	0.0030±0.0020	0.7266	0.3932	0.3452
Pro	1.2789±0.1242	1.3274±0.5428	0.9276±0.1379	1.3355±0.7179	0.4177	0.5970	0.5206

* Abbreviations: BD, Basal diet; HF, High fat diet; BDM, Basal diet +3% monosodium L-glutamate; HFM, High fat diet +3% monosodium L-glutamate.

**Table 10:** Effect of dietary fat and MSG and their interaction on free AA concentrations in the ileum of growing pigs (n = 8).

AA	Measurements (Mean±SD) µmol/g				P values		
	BD*	HF*	BDM*	HFM*	Fat Effect	GSM Effect	Interaction
Arg	0.6939±0.1130	0.8109±0.0868	0.5401±0.0849	0.7874±0.1477	0.0066	0.1364	0.2635
His	0.3316±0.0764	0.3551±0.0629	0.2598±0.0732	0.3550±0.0679	0.1168	0.3264	0.3276
Ile	0.3233±0.0412	0.2986±0.0694	0.2358±0.0268	0.3085±0.1092	0.5016	0.2835	0.1846
Leu	0.6796±0.1147	0.7178±0.1602	0.5576±0.0660	0.7690±0.1725	0.0895	0.6095	0.2240
Lys	0.4811±0.1273	0.5067±0.0695	0.3736±0.0412	0.5688±0.1337	**0.0489**	0.6606	0.1183
Met	0.4474±0.1024	0.4372±0.1006	0.3573±0.1155	0.4587±0.1524	0.4604	0.5765	0.3687
Phe	0.9364±0.0599	0.8970±0.1574	0.7045±0.1840	1.0945±0.1342	**0.0292**	0.8123	**0.0104**
Thr	0.4689±0.0866	0.5389±0.0951	0.5555±0.1901	0.4996±0.1241	0.9156	0.7231	0.3534
Trp	0.8627±0.1473	0.9510±0.2068	0.7798±0.1448	0.9439±0.2370	0.2047	0.6409	0.6941
Val	0.4841±0.1224	0.4654±0.1393	0.3896±0.0841	0.5294±0.1290	0.3347	0.8042	0.2127
Gly	7.7682±1.1321	9.6239±2.3436	7.8509±1.8660	10.2757±1.5031	**0.0323**	0.6853	0.7532
Ser	0.8233±0.1116	0.9518±0.1303	0.7509±0.1010	1.0095±0.2147	**0.0214**	0.9218	0.3918
Tau	0.0072±0.0022	0.0059±0.0010	0.0072±0.0013	0.0076±0.0016	0.5656	0.3056	0.3315
Tyr	0.4332±0.0794	0.4714±0.0981	0.3451±0.0735	0.4228±0.0760	0.1843	0.1225	0.6388
Asn	0.6599±0.0734	0.7309±0.2857	0.5373±0.0880	0.7789±0.1832	0.1065	0.6849	0.3595
Asp	0.2788±0.0617	0.2869±0.0982	0.2584±0.0279	0.3670±0.0816	0.1323	0.4249	0.1894
Cit	0.1565±0.0804	0.1267±0.0232	0.1522±0.0835	0.1610±0.0445	0.7452	0.6443	0.5527
Glu	1.0496±0.1825	1.1395±0.2393	0.9287±0.1621	1.1383±0.2880	0.2050	0.5946	0.6017
Gln	3.2146±0.6169	3.2173±0.4911	2.3496±0.1585	3.6809±0.9247	0.0502	0.5248	0.0510
Orn	0.2292±0.0296	0.2496±0.0671	0.2010±0.0610	0.2454±0.0949	0.3556	0.6388	0.7276
Cys	0.5306±0.3165	0.6121±0.3135	0.5298±0.1606	0.7811±0.4243	0.3157	0.6063	0.6033
Hcy	0.0222±0.0056	0.0237±0.0017	0.0218±0.0035	0.0265±0.0066	0.2149	0.641	0.5101
Abu	0.3001±0.0747	0.3332±0.1107	0.2867±0.0425	0.2969±0.0296	0.5561	0.5003	0.7542
Ala	1.4250±0.2814	1.3568±0.2125	0.8878±0.1514	1.3909±0.2018	0.0679	**0.0387**	**0.0218**
Car	0.0031±0.0036	0.0019±0.0039	0.0029±0.0034	ND	-	-	-
Hyp	1.4200±0.1535	1.4909±0.2934	1.2992±0.1903	1.1077±0.3018	0.6291	0.0606	0.3022
1MHis	0.0573±0.0154	0.0686±0.0116	0.0559±0.0140	0.0825±0.0247	**0.0469**	0.4834	0.3885
3MHis	0.0017±0.0013	0.0023±0.0016	0.0018±0.0013	0.0021±0.0015	0.5589	0.9419	0.8053
Pro	0.9811±0.1774	1.1048±0.2707	0.9695±0.2947	1.1710±0.1952	0.1997	0.8240	0.7511

* Abbreviations: BD, Basal diet; HF, High fat diet; BDM, Basal diet +3% monosodium L-glutamate; HFM, High fat diet +3% monosodium L-glutamate.

## Discussion

Dietary fat and protein are two of the main macronutrients required for life. Many published work had tested the effects of each nutrients independently, however, a few studies are available on the effect of combination of dietary fat and protein. The metabolism is a systemic network, and it would be misleading to evaluate the effects of one nutrient while ignoring the effects of others in plausible nutritional situation. As early as 1956, Mellinkoff et al. found that a diet that contained very few short-chain saturated fats and relatively abundant long-chain unsaturated fats could significantly reduce Leu and Val and increase Pro, Cys, Arg and Asp in plasma [15]. Dietary fatty acids, particularly n-3 fatty acids, can spare AAs for protein and peptide synthesis [16]. On the contrary, AAs, especially those containing sulfur, can also modulate lipid metabolism [17]. As the Chinese economy is developing, the Chinese population, including children and adolescents, has undergone a rapid transition to a high-fat diet [6]. In parallel with these trends, there has recently been a large increase in the consumption of MSG as an umami food additive in China. MSG can facilitate the gastric emptying of a protein-rich meal, and plays an important role in protein digestion [18]. Conversely, MSG has been shown to increase the stomach antral area in human volunteers fed with a normo-proteic diet [19] and to slow gastric emptying in preterm piglets [20]; suggesting that the effects of MSG on the stomach physiology depends on nutritional conditions. In the present study, the effects of dietary fat and MSG on AA metabolism in growing pigs were determined, along with the interaction between these two factors.

MSG begins to release Glu and sodium in the mouth, while dietary fat begins to be digested in the small intestine. As the alimentary bolus rapidly passes through the duodenum, with dietary fat having little effect there, an increase in the luminal concentration of MSG may be responsible for the reduced expression of EAAC1 in the small intestine mucosa. This may correspond to a regulation of AA transport through the enterocytes. Glu released from MSG, is an important precursor for bioactive molecules, including glutathione (oxidative stress modulator) [21], and this may be related to the down-regulation of PEPT1. However, the mechanisms responsible for the antagonistic effects of dietary fat and MSG on B^o+^ remains unclear.

Colonization of the gastrointestinal tract by gut microbiota can influence the absorption and metabolism of nutrients, including AAs. Gut microbiota can incorporate and degrade some available AAs, and can also synthesize AAs [22]; even if the net result of these catabolic and anabolic pathmays remains unknown [22]. Factors that alter the composition of the gut microbiota can also disturb AA metabolism. There have been reports showing that both dietary fat [23], [24] and MSG can influence the composition of the gut microbiota. In the present study, changes in microbiota composition following dietary fat and MSG ingestion were observed [unpublished data]. Changes in the gut microbiota may participate in the up-regulated expression of AA transporters in the jejunum, but further experiments are required to test this hypothesis. Glu is the main oxidative fuel for the gut microbiota in the upper gastrointestinal tract [25]. Even when Glu is provided in higher (4-fold more) amount than normal dietary quantities, most glutamate molecules are either oxidized or metabolized by the mucosa into other nonessential AAs [26]. Thus, both dietary fat and MSG may enhance AA synthesis by the gut microbiota, and this may be related to the effects of dietary fat and MSG on the expression of AA transporters. In the lower gastrointestinal tract, the effects of dietary fat and MSG were weak, and this is presumably related to the higher density of bacteria in these two intestinal segments than in the jejunum, and to the fact that AAs degraded from the residual undigested luminal proteins and peptides cannot be absorbed in the distal intestine [22].

The kidney is an important organ that finely tune the circulating and tissue pools of AAs. Consistent with previous findings that protein intake, in contrast to fat and carbohydrate, can significantly influences renal hemodynamics in healthy animals and humans [27], [28], dietary fat had little effect on the AA profile and could only significantly lower L-carnosine (Car) levels in the kidney were found [29], but no similar change of Car was seen in the liver. In contrast, supplementation with MSG may disturb the renal AA balance and increase the AA pool in the kidney. If glutamate which has not been metabolized by the intestinal mucosa is transported to the kidney, it can be converted to glutamine [30] and then further transformed to other non-essential AAs such as Ser, Tyr and Arg, and exported to other tissues [31]. Unexpectedly, some essential AAs, like His, Ile, Leu, Met, Thr and Val, were also increased, which may be due to the effect of glutamate on the kidney [32], and/or to the sparing effect of glutamate on the utilization of several AAs in tissues including the intestinal mucosa [19]. Indeed, MSG is able to increase Leu in the kidney and muscle [33]–[35]. Another notable change is the increase of Cit in the kidney. Cit is a metabolic substrate for mutual conversion with Arg [36], the latter AA plays multiple roles: it is a precursor of nitric oxide, creatine, agmatine, and other polyamines, and is an intermediate in the urea cycle [37]–[39]. In the present study, Gly was elevated by dietary fat, and this may be related to the fact that fat can reduce the oxidation of AAs.

Under a condition of brief caloric restriction, dietary fat had no effect on the conservation of body proteins and branched-chain AAs, including Leu, Ile and Val [40]. However, under normal conditions, supplementation with dietary fat can reduce the percent of energy provided by other nutrients such as AAs. The results also showed that fat can lower serum levels of glucogenic AAs. Serum Val is a good predictor [41], and serum Val was increased induced by supplementation of dietary fat was found. The results suggest that supplementation with dietary fat can promote the retention of AAs.

An increased level of serum 3MHis in cattle is considered as a measurement of muscle protein degradation [42]. MSG was able to significantly lower serum 3MHis, which indicates that MSG may inhibit protein degradation in muscle, but more experiments might be required to test this hypothesis.

In the present study, the expression of AA-sensing genes and AA transporters in tissues were also measured. While the determination of AA-sensing genes are likely indirectly related to AA concentrations, the results obtained are compatible with the view that AA concentration and gene expression in tissues may influence each other in both directions.

## Conclusion

In conclusion, the effects of dietary fat and MSG, both alone and in combination, on the AA in the circulating and tissue pool were determined. Little interaction was found between the effects of dietary fat and MSG. Both dietary fat and MSG enhanced the absorption of AAs in jejunum. Dietary fat can enhance the AA pool in plasma and muscle, while MSG enhances AA pool in the kidney. The results of the present study will help to further uncover the effects of dietary fat and MSG addition on human amino acid metabolism, with predictable consequences for the optimization of animal feeding and human nutrition.
